# The Convergence of the Hedgehog/Intein Fold in Different Protein Splicing Mechanisms

**DOI:** 10.3390/ijms21218367

**Published:** 2020-11-07

**Authors:** Hannes M. Beyer, Salla I. Virtanen, A. Sesilja Aranko, Kornelia M. Mikula, George T. Lountos, Alexander Wlodawer, O. H. Samuli Ollila, Hideo Iwaï

**Affiliations:** 1 Institute of Biotechnology, University of Helsinki, P.O. Box 65, FIN-00014 Helsinki, Finland; hannes.beyer@uni-duesseldorf.de (H.M.B.); salla.i.virtanen@helsinki.fi (S.I.V.); sesiljaaranko@gmail.com (A.S.A.); kornelia.mikula@helsinki.fi (K.M.M.); samuli.ollila@helsinki.fi (O.H.S.O.); 2 Basic Science Program, Frederick National Laboratory for Cancer Research, Frederick, MD 21702, USA; lountosg@mail.nih.gov; 3 Macromolecular Crystallography Laboratory, National Cancer Institute, Frederick, MD 21702, USA; wlodawer@nih.gov

**Keywords:** protein-splicing mechanism, intein, protein engineering, protein evolution, protease

## Abstract

Protein splicing catalyzed by inteins utilizes many different combinations of amino-acid types at active sites. Inteins have been classified into three classes based on their characteristic sequences. We investigated the structural basis of the protein splicing mechanism of class 3 inteins by determining crystal structures of variants of a class 3 intein from *Mycobacterium chimaera* and molecular dynamics simulations, which suggested that the class 3 intein utilizes a different splicing mechanism from that of class 1 and 2 inteins. The class 3 intein uses a bond cleavage strategy reminiscent of proteases but share the same Hedgehog/INTein (HINT) fold of other intein classes. Engineering of class 3 inteins from a class 1 intein indicated that a class 3 intein would unlikely evolve directly from a class 1 or 2 intein. The HINT fold appears as structural and functional solution for *trans*-peptidyl and *trans*-esterification reactions commonly exploited by diverse mechanisms using different combinations of amino-acid types for the active-site residues.

## 1. Introduction

**Figure 1:**
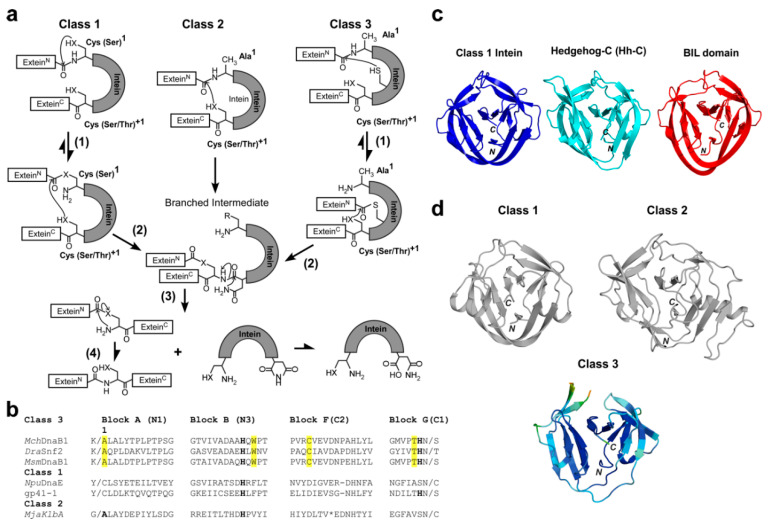
Protein splicing reaction steps for class 1, 2, and 3 inteins [8,9,12,14]. (**a**) Protein splicing mechanisms for class 1 inteins with four concerted steps: (1) N–X acyl shift (X = O or S); (2) *Trans*-(thio)-esterification; (3) Asn cyclization; (4) X–N acyl shift (X = O or S), for class 2 inteins: (2) *Trans*-(thio)-esterification; (3) Asn cyclization; (4) X–N acyl shift (X = O or S), and for class 3 inteins: (1) Thio-ester formation; (2) *Trans*-(thio)esterification; (3) Asn cyclization; (4) N-X acyl shift (X = O or S). (**b**) A sequence alignment of class 1, 2, and 3 inteins for blocks A, B, F, and G. Highly conserved His residues in blocks B and G are shown in bold. The characteristic Ala at the first residue of the class 2 intein is in bold. (**c**) Ribbon drawing of the structures of representative HINT (Hedgehog/INTein) superfamily members: *Npu*DnaB class 1 intein (4o1r) [15], the C-terminal domain of the hedgehog protein (Hh–C, 1at0) [10], and Bacterial Intein-Like (BIL) domain (2lwy) [16]. (**d**) The crystal structure of class 3 *Mch*Dna1 intein and representative class 1 and 2 inteins. The ribbon drawing of the class 2 intein is based on the *Mja*KlbA intein (2jnq) [17], and the class 1 intein on the *Npu*DnaE intein (4kl5, chain A) [18]. The ribbon drawing of the *Mch*Dna1 intein (6rix, chain B) structure is colored according to the temperature factor. *N* and *C* denote the N- and C-termini, respectively.

Protein splicing is catalyzed by intervening protein sequences termed inteins. The protein-splicing reaction involves the self-removal of the intein and concomitant joining of the two flanking sequences (exteins) (Figure 1) [1,2]. Protein splicing is analogous to RNA splicing but occurs on the protein level. The biological function of protein splicing is still enigmatic despite several proposals for eventual regulatory functions [3]. Inteins are often considered merely as selfish gene elements because they can be generally removed without any fitness cost for their host organisms. Inteins commonly insert in conserved sequences close to the active sites of essential proteins. Any mutations within inteins detrimental to the protein splicing activity could be lethal or strongly affect the fitness of their host, thus likely ensures intein persistence and protection from functional degeneration during evolution [4].

Over 1500 inteins have been identified based on the characteristic conserved amino-acid sequences defined as the N- and C- terminal intein motifs (blocks, A, B, F, and G in Figure 1b) [5,6,7]. The most common protein splicing mechanism has been generally accepted and involves four concerted steps: (1) N–S(O) acyl shift between the immediately preceding peptide bond and Cys1 (or Ser1), (2) *trans*-(thio)esterification, (3) Asn cyclization, and (4) S(O)–N acyl shift to form an energetically favorable peptide bond (Figure 1a) [8]. Inteins catalyzing the canonical splicing mechanism are referred to as class 1 inteins (Figure 1a,b) [9]. All splicing domains found among inteins have the same structural architecture named HINT (Hedgehog/INTein) which relates to the C-terminal domain of the Hedgehog protein (Hh–C or hog domain). However, not all of the four steps are exploited among all HINT superfamily members, including those catalyzing reactions related to protein splicing such as bond cleavage (Figure 1c) [2,10]. For example, the C-terminal domain of the Hedgehog protein (Hh–C or hog domain), the eponymous member of the HINT superfamily, uses only the initial N–S acyl shift for cholesterol modification of the N-terminal signaling domain (Hh–N) [2,10]. Bacterial Intein-Like (BIL) domains lack the nucleophilic +1 residue common among most inteins which is essential for the *trans*-esterification step in the protein-splicing reaction and therefore produce predominantly cleaved products [11]. Some inteins do not catalyze the canonical splicing reaction of class 1 inteins. Inteins lacking the first nucleophilic residue (Cys1 or Ser1) required for the initial N–S(O) acyl shift step were originally termed class 2 inteins (Figure 1a,b) [12]. However, class 3 inteins lacking the N-terminal serine or cysteine, similar to class 2 inteins, have been identified due to the conserved Trp–Cys–Thr (WCT) motif found only among class 3 inteins (Figure 1b) [13,14]. Instead of the N-terminal serine or cysteine missing among class 3 inteins, class 3 inteins contain an additional nucleophilic cysteine residue in block F (Figure 1b). The cysteine in block F is part of the unique WCT motif and substitutes the function of the N-terminal nucleophilic residue of class 1 inteins required for the initial acyl shift step (N–X acyl shift, Figure 1a) [9,13,14]. Class 3 inteins are thus classified as a distinct class of inteins from class 2 inteins (Figure 1a,b).

Whereas the first residue of class 1 inteins can be cysteine or serine, the C-terminal nucleophilic residue at the +1 position of inteins is usually either cysteine, serine, or threonine (Figure 1a). Although the penultimate histidine residue and histidine residue in block B are highly conserved among many inteins (Figure 1b), several inteins lack these histidine residues and remain capable of catalyzing protein splicing due to compensatory mutations [19,20]. Inteins catalyzing protein splicing are thus unique single-turnover enzymes that tolerate high sequence variations at the active site residues even among the same class of inteins. Thus, inteins do not have strict requirements for the active site residues but utilize slightly different protein-splicing mechanisms by compensating mutations.

The current notion in the field suggests that members of the HINT superfamily have evolved from a common ancestor by divergent evolution [9]. Although the HINT fold can be easily detected based on the sequence homology, significant deviations of the active-site-residue combinations at all critical residues have also been observed [5,6,13].

In this work, we asked how inteins evolved with different splicing mechanisms despite the low sequence conservation and high variation of the catalytic residues. We addressed these questions by elucidating the structural basis for the protein splicing mechanism of class 3 inteins by crystal structures, molecular dynamics simulations, and structure-based protein engineering.

## 2. Results

**Figure 2:**
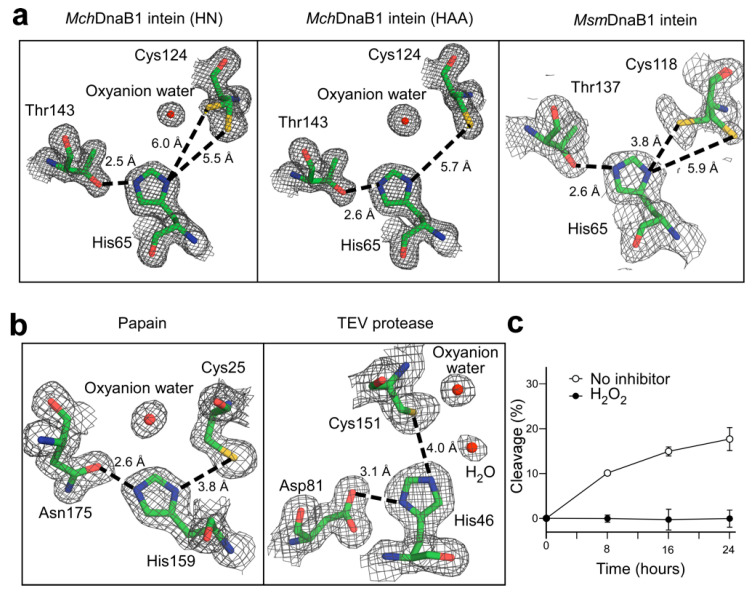
Comparison of the active sites. (**a**) Comparison of the electron density maps at 1σ counter level around the catalytic-triad between the class 3 inteins: *Mch*DnaB1_HN (6rix, chain B), *Mch*DnaB1_HAA (6riy, chain A), and *Msm*DnaB1 inteins (6bs8, chain B) [21]. Oxyanion waters are modeled for the large electron density near Cys124. Dashed lines indicate distances between Oγ of Thr and Nε of His and between Sγ of Cys and Nδ of His. When two conformations for Cys were modelled, we showed both distances. (**b**) The electron density maps of the catalytic triad from papain (1ppn) and TEV protease (1lvm, chain A). The catalytic triads are depicted together with the electron density maps at 1.1 sigma counter level. Dashed lines indicate the shorter distances between Oδ of Asn or Asp and site-chain N atoms of His and between Sγ of Cys and side-chain N atoms of His. (**c**) Inhibition of the N-cleavage of *Mch*DnaB1_HN by H_2_O_2_. The data were averaged from three replicates. Error bars represent one standard deviation.

To gain a better understanding of the class 3 intein splicing mechanism, we decided to obtain three-dimensional structures. We originally attempted the crystallization of the class 3 DnaB1 intein from *Mycobacterium smegmatis* (*Msm*DnaB1) but failed, presumably because the purified *Msm*DnaB1 intein was not well-folded as judged from the HSQC spectrum (Supplemental Figure S1b). This observation was in line with our tests for protein *cis*-splicing activity of class 3 inteins from *Deinococcus radiodurans* (*Dra*), *Mycobacterium smegmatis* (*Msm*), and *Mycobacterium chimaera* (*Mch*) using a model protein system. We selected *Mch*DnaB1 intein, because it is relatively small and showed a high protein splicing activity at 37 °C as judged from the amount of spliced product after purification among the three class 3 inteins tested (Supplemental Figure S1). We determined the high-resolution crystal structures of two variants of the class 3 *Mch*DnaB1 intein (*Mch*DnaB1_HN and *Mch*DnaB1_HAA; Figure 2a and Supplemental Figure S2). *Mch*DnaB1_HN (1.66 Å resolution) lacked the C-terminal extein sequence, whereas *Mch*DnaB1_HAA (1.63 Å resolution) contained a C-terminal extein residue (Ala) at the +1 position (Ser+1Ala) and a mutation of the terminal Asn residue to Ala (Asn145Ala) (Figure 1c, Supplemental Figure S2 and Supplemental Table S1). The *Mch*DnaB1 intein structure shares the typical HINT fold of class 1 and class 2 inteins, which is in line with the previous report of a class 3 intein structure (Figure 1b,c) [10,21]. Thus, the class 3 *Mch*DnaB1 intein is indistinguishable from class 1 and class 2 inteins when comparing their backbone conformations, because additional insertions and deletions observed among inteins easily mask their differences (Figure 1c) [22]. We found that the most striking feature of the crystal structures of the *Mch*DnaB1 intein is the active site, closely resembling the catalytic triad of serine/cysteine proteases. The observed distance (5.5–5.7 Å) between Sγ atom of Cys124 and Nδ atom of His65 in the *Mch*DnaB1 inteins is slightly longer than in typical cysteine proteases (3.8–4.0 Å) (Cys25 and His159 for papain and Cys151 and His51 for TEV protease) (Figure 2a,b) [23]. The WCT motif found in the class 3 intein participates in forming the catalytic triad, in which Cys124, His65, and Thr143 could serve as nucleophilic, basic, and acidic functional groups, respectively (Figure 2a,b). Importantly, we could observe clear electron density near the side-chains of Cys124, His65, and the backbone of Val125 for both crystal structures of the *Mch*DnaDB1_HN and *Mch*DnaB1_HAA inteins (modeled as oxyanion waters in Figure 2a and Supplemental Figure S2). This electron density could be the oxyanion hole that is commonly observed in the crystal structures of serine/cysteine proteases, stabilizing the tetrahedral reaction intermediate (Figure 2a,b) [23]. In the class 3 intein structure, Thr143 in block G serves as the protonating acidic residue instead of aspartic acid in the typical Ser-His-Asp catalytic triad of serine proteases. The weaker acidity of Thr compared to Asp might lower not only the nucleophilicity of Cys124 but also increase the distance between His65 and Cys124. However, inteins are single turnover enzymes requiring only one splicing reaction per molecule, rendering high reactivity redundant. Thus, the Cys-His-Thr catalytic triad in *Mch*DnaB1 intein could be sufficient for creating the acyl-enzyme intermediate similar to that found in many serine/cysteine proteases as previously suggested [24].

### 2.1. Self-Cleavage Activity and Inhibition of Class 3 Inteins by Protease Inhibitors

Both variants of the *Mch*DnaB1 intein were produced for crystallization as N-terminal SUMO fusion proteins, resulting in the N-terminal “SVGK” extein sequence after Ulp1 protease treatment to remove the SUMO fusion tag. However, the crystal structures of both *Mch*DnaB1 intein variants (HN and HAA at the C-terminus) lacked electron densities for the N-extein sequences. This observation is presumably due to self-cleavage at the N-terminus during sample preparation and/or crystallization (N-cleavage) [21]. We also confirmed the N-cleavage activity in vitro by incubating the freshly purified fusion proteins (Supplemental Figure S3). As observed for other class 3 inteins, a mutation of the last Asn145 residue to Ala in the *Mch*DnaB1 intein (*Mch*DnaB1_HAA) largely halted the reaction at the branched acyl-intein intermediate (Supplemental Figure S3c). Assuming a protease-like mechanism, we tested the inhibition of N-cleavage using common inhibitors of cysteine proteases, phenylmethanesulfonyl fluoride (PMSF) and oxidizing reagent hydrogen peroxide (H_2_O_2_) as well as protease inhibitor cocktails (Figure 2c and Supplemental Figure S3b–d) [25,26]. Whereas PMSF had little statistically significant effect on N-cleavage, H_2_O_2_ showed clear inhibition (Figure 2c and Supplemental Figure S3b–c). Due to its small size, H_2_O_2_ could easily access to the oxyanion hole, thereby oxidizing Cys124, while PMSF may be sterically-hindered in accessing the active-site cysteine residue due to the larger 140 Å^3^-molecular volume [27], as inteins process an intramolecular substrate. These observations corroborate the notion that a class 3 intein might utilize a catalytic triad similar to serine/cysteine protease for producing the acyl-enzyme intermediate. While most inteins generally auto-catalytically splice immediately after protein translation, the mini-chromosome maintenance protein 2 intein from *Halorhabdus utahensis* (*Hut*MCM2) is inactive at a low salt concentration but can be activated with high salt concentrations [28]. To further verify the class 3 splicing mechanism, we used the salt-inducible *Hu*tMCM2 intein for testing the effect of H_2_O_2_ on the N-cleavage of a class 1 intein in an in vitro model [28]. We found that H_2_O_2_ did not inhibit N-cleavage of the salt-inducible class 1 intein at a high salt condition, further supporting the protease-like acyl-enzyme intermediate for the class 3 splicing mechanism (Supplemental Figure S4).

### 2.2. Conversion of a Class 1 Intein into a Class 3 Intein

Previously, conserved active site mutations among the HINT superfamily were used to demonstrate evolutional connections. For example, BIL domains that predominantly produce N- and C-cleaved instead of spliced products were converted into very efficient protein splicing domains by a single mutation. This observation suggested that BIL domains divergently evolved from an ancestral intein [11,16]. Likewise, class 2 inteins lacking Ser or Cys at the N terminus could also efficiently splice after the replacement of Ala at the +1 position by Cys or Ser, suggesting a clear evolutionary connection to class 1 inteins [12].

**Figure 3:**
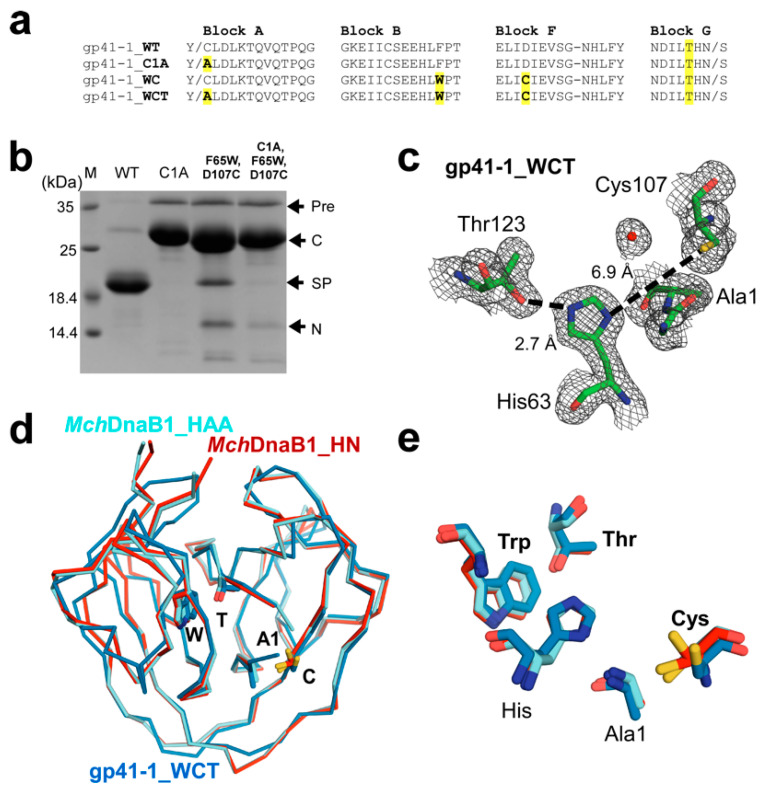
Conversion of a class 1 into a class 3 intein by grafting the WCT motif. (**a**) Sequence alignment of the engineered variants of the gp41-1 intein with different mutations. The WCT motif and Cys1Ala substitution are highlighted in yellow. (**b**) SDS-PAGE analysis of protein *cis*-splicing by the engineered variants with indicated mutations. M, molecular weight marker; WT, wild-type; Pre, precursor; C, C-cleavage product; SP, splicing product; N, N-cleavage product. (**c**) A close-up of the electron density map observed for the active site of the WCT motif-grafted class 1 intein, gp41-1_WCT with the same orientation as the structures shown in Figure 2a. (**d**) Superposition of the three crystal structures of the gp41-1 intein with the WCT motif (gp41-1_WCT) (blue), *Mch*DnaB1_HN (red), and *Mch*DnaB1_HAA (cyan). The residues of the WCT motif are shown by stick models and indicated, together with the first residue of the inteins. (**e**) A close-up of the WCT motif together with His in the catalytic triad from the superposition of the three structures shown in (**d**).

We decided to use the same strategy for testing the divergent evolution model of class 3 inteins from class 1 inteins as previously demonstrated with class 2 intein and BIL domains [16,29]. We assumed that introducing the unique WCT motif found in class 3 inteins into a class 1 intein together with the first Cys/Ser to Ala mutation could possibly result in a functional *cis*-splicing intein if they were closely related by a divergently evolved lineage, similar to class 2 and BIL domains. We chose the class 1 gp41-1 intein as a template intein because gp41-1 intein has a Thr residue at the corresponding position of the WCT motif of class 3 inteins, and the 1.0 Å-resolution crystal structure (6qaz) is available, facilitating the WCT motif engineering [30]. We introduced the WCT motif on the gp41-1 intein based on the amino-acid sequence alignment (Figure 3a and Supplemental Figure S1a). However, the engineered class 3 gp41-1 intein (gp41-1_WCT) produced dominantly the C-cleaved product and only a minute amount of the possible splicing product (Figure 3b). This result indicates that class 3 intein requires additional compensatory mutations in addition to the WCT motif for productive protein splicing. To better understand the structural basis for non-productive splicing of the engineered class 3 intein, we solved the crystal structure of gp41-1_WCT at 1.85 Å resolution (Figure 3c–e). Unlike in the crystal structures of *Mch*DnaB1_HAA and *Mch*DnaB1_HN, we observed electron density for the N-terminal extein, confirming that gp41-1_WCT is inactive in proteolytic cleavage at the N-terminal junction (N-cleavage). The catalytic triad of Cys124-His65-Thr143 and Trp67 from the WCT motif in the *Mch*DnaB1 intein can be precisely superimposed with the engineered triad of Cys107-His63-Thr123 and Trp65 (0.39 Å for the r.m.s.d. was obtained for the 35 heavy atoms of these four residues excluding Sγ of Cys124), except for the χ^1^ angle of the nucleophilic Cys107 (Figure 3d,e). The presence of the N-extein (see below) likely induced the *trans* conformation of Cys107 in gp41-1_WCT. Despite successful engineering of the critical WCT motif on the structure of gp41-1_WCT to mimic *Mch*DnaB1 intein, gp41-1_WCT mainly resulted in non-productive cleavages without any protein-splicing product (Figure 3b). The unsuccessful conversion of a class 3 intein contrasts with the results from the engineering of a class 2 intein and BIL domains into class 1-like inteins, in which simple mutations created protein-splicing active variants. This reverse engineering suggests that class 3 inteins require additional compensatory mutations in addition to the WCT motif to be proficient in protein splicing. Such simultaneous compensatory mutations on class 1 or 2 inteins together with the WCT motif is an improbable evolutionary event according to the current survival model of inteins, which are usually inserted near the active site of enzymes essential for host organisms [2,4]. A plausible alternative explanation for the emergence of class 3 inteins is that they have gone through a unique evolutionary pathway different from other HINT members.

### 2.3. The Active Site of the MchDnaB1 Class 3 Intein

Despite sharing the same HINT fold, class 3 inteins appear to utilize a very different mechanism for the same protein splicing reaction compared to other members of the HINT superfamily [8,10,12,14]. Available intein structures containing the extein sequences, except for the two coordinate sets of *Sce*VMA and *Pho*RadA inteins, typically have large distances (~8–9 Å) between the N-scissile peptide and the nucleophilic side chain of the +1 residue responsible for the second reaction step, namely *trans*-esterification [31,32,33]. These longer distances suggest the necessity of substantial conformational changes for class 1 inteins during protein splicing. We observed electron density for both the *gauche*+ and *trans-like* conformations of Cys124 in the crystal structure of *Mch*DnaB1_HN, although the side-chain conformation of Cys124 in the *trans-*like conformation is less evident in the second molecule (chain B) in the asymmetric unit (Figure 2a and Supplemental Figure S2). A similar alternative conformation was also reported for the structure of another class 3 intein, the DnaB1 intein of *Mycobacterium smegmatis* (*Msm*DnaB1 intein) (Figure 2a) [21]. On the other hand, the variant of *Mch*DnaB1_HAA shows overall weaker densities for the second conformation in *gauche*+ for Cys124, which was not modeled (Figure 2a). In the *Mch*DnaB1_HAA intein bearing an extein residue, the distance between the Cβ atom of the +1 residue (Ala) and Sγ atom of Cys124 is 4.7–5.0 Å. However, this distance with the +1 residue of the C-extein would be much shorter (<3.0 Å) when the χ^1^ angle of Cys124 was in the *trans* conformation. The rotation of the χ^1^ angle of Cys124 could thus bring the nucleophilic atom sufficiently closer to the +1 residue, promoting the *trans*-esterification reaction step without requiring the substantial conformational changes reported for other class 1 intein structures [31,32,33]. Therefore, we believe that the rotamer of Cys124 could play an essential role in the splicing reaction of class 3 inteins, which differs from the reported large conformational changes in the reaction mechanisms of class 1 and 2 inteins [24,31,32,33].

### 2.4. Molecular Dynamics Simulation

**Figure 4:**
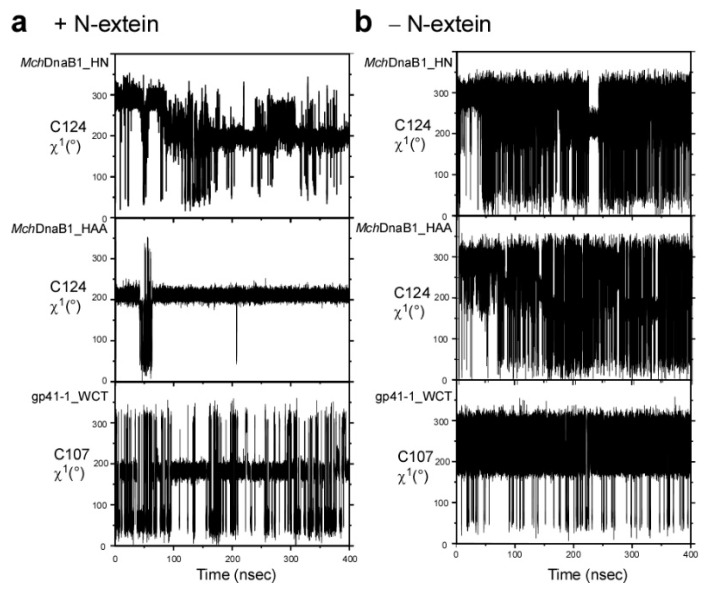
Analysis of χ^1^ angles of Cys in the WCT motif during 400-nsec MD simulations of the two variants of the *Mch*DnaB1 intein (*Mch*DnaB1_HN and *Mch*DnaB1_HAA) and the engineered gp41-1 intein with WCT motif (gp41-1_WCT). (**a**) Trajectories of the χ^1^ angle for the cysteine residues in the WCT motif during the 400-nsec MD simulation with the modeled N-extein sequence. (**b**) Trajectories of the same χ^1^ angle without N-extein. The same analysis for other residues of the catalytic triad are show in Supplemental Figure S6.

**Figure 5:**
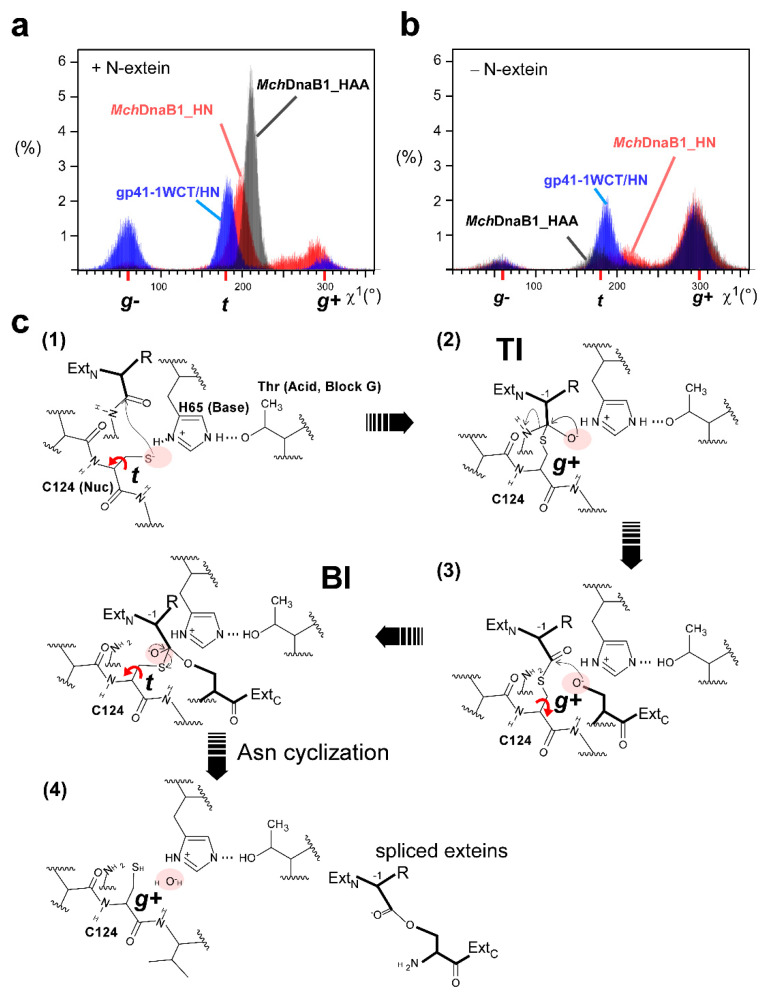
MD simulations and the proposed splicing steps by class 3 inteins. Histograms showing the distributions of the χ^1^ angle for the cysteine residue in the WCT motif during the MD simulations of the two *Mch*DnaB1 intein variants and the engineered gp41-1 intein with WCT motif with the modeled N-extein (**a**) and without N-extein (**b**). Red, grey, and blue indicate the population data for *Mch*DnaB1_HN, *Mch*DnaB1_HAA, and gp41-1_WCT, respectively. (**c**) Proposed reaction steps for the protein-splicing mechanism by the class 3 intein. (1) High energy ground state before splicing with Cys124 in the unfavorable *trans*-like conformation. (2) Tetrahedral Intermediate (TI) status after rotation of Cys124 to the gauche^+^ conformation to favor the nucleophilic attack. (3) Formation of the Branched Intermediate (BI) after N-cleavage. Rotation of Cys124 to the *trans* conformation will bring the thioester intermediate closer to the nucleophilic residue of the C-extein. *Trans*-esterification step via a tetrahedral intermediate. Rotation of Cys124 back to the gauche+ conformation. (4) Post-splicing status. Exteins are released from the intein. A red arrow indicates a rotational movement of Cys124. Pink shadows indicate possible locations for oxyanion holes. See main text for a more detailed description.

To support our interpretation of the *Mch*DnaB1 intein crystal structures, we performed 400-nanosecond molecular dynamics (MD) simulations of *Mch*DnaB1_HN, *Mch*DnaB1_HAA, and the engineered gp41-1_WCT in the presence or absence of the four-residue N-extein. We observed noteworthy differences between the different MD simulations with and without the modeled N-extein for the side-chain conformation of Cys124. The presence of the modeled N-extein pushed the side-chain rotamer of Cys124 in both *Mch*DnaB1_HN and *Mch*DnaB1_HAA structures towards the less favorable *trans*-like conformation (χ^1^ = ~200–210°) (Figure 4 and Figure 5). Upon removal of the N-extein in the simulation, the population largely shifted towards the ideal *gauche*+ conformation with χ^1^ = ~300° (−60°), with more frequent rotation between *gauche*+ and *trans*-like conformations (Figure 4 and Figure 5). This observation might suggest that both crystal structures represent the post-splicing or post-cleavage status as expected from the primary structure of the variants (Supplemental Figure S5). Interestingly, the MD simulations also revealed distinct differences between the engineered gp41-1_WCT and *Mch*DnaB1 intein variants. Among the three inteins used in the MD simulations, gp41-1_WCT with the N-extein showed the most abundant population for *gauche*- and the χ^1^ angle of the introduced Cys107 was much closer to the ideal 180°-*trans* conformation than to ~200–210° observed in the other simulations for the two *Mch*DnaB1 inteins (Figure 4 and Figure 5a,b). This energetically less favorable *trans*-like conformation observed in the *Mch*DnaB1 intein variants might suggest that it could be a driving force for the splicing reaction in class 3 inteins.

### 2.5. The Catalytic Mechanism of Class 3 Inteins

Based on biochemical and structural data as well as MD simulations, we propose the catalytic mechanism of class 3 inteins, as depicted in Figure 5c. At the pre-splicing state, Cys124 is in the high-energy (unfavorable) *trans*-like conformation (χ^1^ = 200°–210°) and weakly deprotonated by His65. The rotation around the χ^1^ angle of Cys124 to *gauche*+ from the high-energy state would induce the first step of the nucleophilic attack and form the tetrahedral intermediate (TI), which is supposedly stabilized by the oxyanion hole. Subsequent N-cleavage creates a thioester bond in the branched intermediate (BI). The rotation of the χ^1^ angle would bring the branched intermediate bearing the thioester bond closer to the nucleophilic oxygen atom of Ser at the +1 position for the *trans*-esterification reaction step via the tetrahedral intermediate that might also be stabilized by the oxyanion hole. The more frequent χ^1^ rotation of Cys124 between the *gauche*+ and *trans*-like conformation in the absence of the N-extein could thus mimic the movement of the branched intermediate bringing the state closer to the +1 residue. The subsequent *trans*-esterification reaction via the tetrahedral intermediate stabilized by the oxyanion hole releases the N- and C-exteins from the intein. The released extein ester will undergo subsequent O–N rearrangement to the energetically favorable peptide bond. Based on our current data, it is unclear whether Asn cyclization will take place before the *trans*-esterification or simultaneously with it. The intein will reach the ground state (*gauche*+ conformation of Cys124), represented by the crystal structure of *Mch*DnaB1_HN. In the absence of the nucleophilic +1 Ser residue, the oxyanion water molecule slowly hydrolyzes the branched intermediate and releases the N-extein. We think that the three-dimensional crystal structure of *Mch*DnaB1_HAA likely represents the post-hydrolysis state of the *Mch*DnaB1 intein (Supplemental Figure S5). In this proposed model for the splicing mechanism of class 3 inteins, the rotational motion of the cysteine in the WCT motif might play a crucial role, unlike in other intein classes where large conformational changes of 8–9 Å are expected to occur for the first N–S(O) acyl shift [31,32,33].

## 3. Discussion

**Figure 6:**
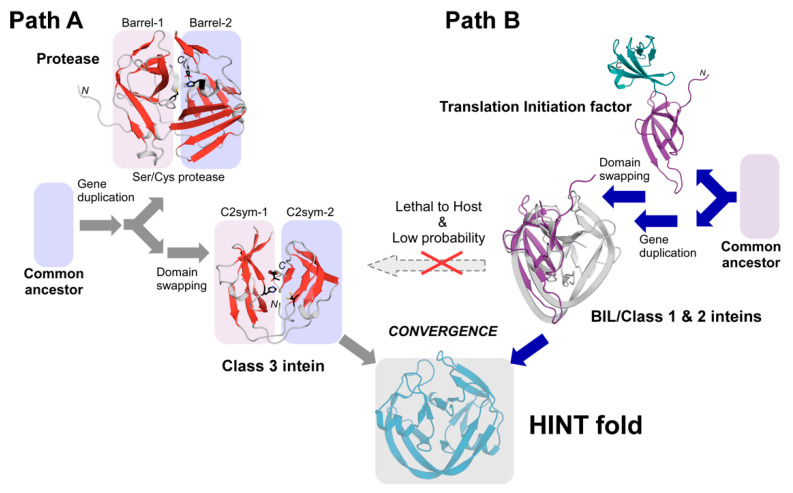
Evolution model of the HINT fold. (**A**) Class 3 inteins may have evolved from an ancestral cysteine protease originating from prophages, retaining the highly conserved catalytic triad. Boxes indicate two sub-domains of a cysteine protease (TEV protease, 1lvm) and the pseudo-C2-symmetry relation in a class 3 intein (*Mch*DnaB1 intein, 6rix). (**B**) Other members of the HINT superfamily might have evolved via a very different pathway from a distantly related ancestral protein such as a translation initiation factor by gene duplication, fusion, and domain swapping. Cartoon drawings of translation initiation factor (IF5A, 1bkb) [41] and the superposition with the pseudo-C2-related subdomain of the BIL domain (2lwy) [16] are shown (Supplemental Table S2). The purple N-terminal domain of IF5A was superimposed with the HINT domain.

One protein fold may serve as a common scaffold for many functions. For example, the eightfold (βα) barrel structure, known as TIM-barrel, is the most common protein fold utilized by many different enzymes with very diverse amino-acid sequences [34]. Whereas a specific protein fold might not be a prerequisite for the function of a protein, the catalytic triad found in proteases is often seen as prime example of convergent evolution [35], because it is very unlikely that two proteins evolve from a common ancestor and retain similar active-site structures while other structural features completely change [36]. Many serine/cysteine proteases, such as chymotrypsin/trypsin, share a common core composed of two-barrel motifs—a result of presumable gene duplication (Figure 6) [37]. The nucleophile-histidine-acid catalytic triad motif of serine/cysteine proteases is located at the interface of the two β-barrels and considered to be the result of convergent evolution. Even though the common horseshoe-like fold of the HINT superfamily members does not have two distinct β-barrels, the HINT fold contains two subdomains related by the pseudo-C2-related symmetry [10,22,30,38]. This symmetry relation may also be the result of gene duplication, fusion, and loop-swapping events [10,37]. The catalytic triad formed by Cys124-His64-Thr143 in the *Mch*DnaB1 intein is analogously split between the two subdomains and located at the interface (Figure 6). As previously suggested, the similarity to proteases [24] arising from the catalytic triad in the *Mch*DnaB1 intein at the interface of the two subdomains of the HINT fold resembles the common catalytic triad of serine/cysteine proteases, including the oxyanion hole stabilizing the tetrahedral intermediate during catalysis (Figure 2a). Since peptide bond formation is the reverse reaction of peptide hydrolysis, it is not surprising that protein splicing uses the same mechanism as cysteine proteases involving a tetrahedral intermediate. Indeed, several peptidases have been used for *trans*-peptidase reactions [39,40].

A comparison between the splicing active *Mch*DnaB1 intein and the WCT motif-engineered non-splicing gp41-1 intein derived from a class 1 intein implies that accumulation of random mutations in a class 1 intein would not directly lead to a class 3 intein. Such a divergent evolution model for class 3 inteins is particularly implausible because any functionally detrimental mutations of the active site residues could reduce the fitness of the host organism or even be lethal. The concurrent occurrence of compensatory mutations to maintain the splicing activity is an improbable event, suggesting that a class 3 inteins cannot directly evolve from a class 1 or 2 intein.

The MD simulations provided additional evidence that the rotational motion of the active-site cysteine could be sufficient for enabling protein splicing of class 3 inteins. Class 3 inteins hence utilize a catalytic mechanism that is different from class 1 and 2 inteins which involve large conformational changes [24,31,32,33]. The WCT motif engineering on a class 1 intein did not lead to similar rotational dynamics of the active site residues, indicating that additional compensatory mutations are necessary for splicing-active class 3 inteins. The structural and biochemical data impose the question of how class 3 inteins could have divergently emerged from class 1 or class 2 inteins. A plausible explanation from the structural basis of the class 3 splicing mechanism could be that class 3 inteins are more distantly related to class 1 and 2 inteins and have evolved from a protease-linage originating from prophages [2,9,14,19,20].

Inteins tolerate a vast array of variations at the active-site residues for protein splicing, leaving the N-terminal Ser or Cys and C-terminal Asn, Gln, or Asp as the only omnipresent amino-acid residues among class 1 inteins. Even the highly conserved histidine in block B and penultimate His is substituted in several inteins [19,42]. These conserved residues can be further reduced to the C-terminal Asn for class 2 inteins, yet retaining the protein splicing activity by different combinations of the catalytic residues and compensatory mutations. One way to explain the extremely high tolerance of the active site residues of inteins is that the HINT fold is the crucial structural solution enabling peptidyl transfer reactions.

In the HINT fold, the enzymes (inteins) and substrates (exteins) are covalently connected as single precursor molecules, thereby working as single-turnover enzymes. Inteins do not require any substrate-association step. The covalent linkage to their substrates could also facilitate the accommodation of different amino-acid types at the active site residues among the HINT superfamily compared with other enzymes. The HINT fold might play a crucial role in bringing the acyl-(thio) ester intermediate and the nucleophilic residue from the C-extein close together, at the precise position and timing required for protein splicing. We gathered evidence suggesting that class 3 inteins might have evolved through a different pathway than class 1 and 2 inteins, possibly related to serine/cysteine proteases originated from prophages because class 3 inteins have a clear monophyletic distribution and an inactive class 3 intein sequence was found within a pseudogene [14,42]. We revisited what might be the possible common ancestral protein of other members among the HINT superfamily. We searched the Protein Data Bank (PDB) using the DALI server with the BIL coordinates (2lwy) [16,43] and identified possible ancestral domains corresponding to the C2-related pseudo-symmetry subdomain in the HINT fold (Supplemental Table S2). Despite their low Z-scores (2.5–2.7), we noticed structural homology to translation initiation factor 5A (1bkb) [41], eukaryotic translation initiation factor 5A2 (3hks) [44], and elongation factor P (1ueb) [45], demonstrating the apparent structural similarity with r.m.s.d. between 1.8 and 2.4 Å for 42–49 residues (Figure 6 and Supplemental Figure S7). Intriguingly, these proteins are also involved in the first step of peptide bond formation in translation utilizing ribosomal protein synthesis. Class 1 and 2 inteins might have descended from a common ancestor shared by translation initiation factors or their ancestor by gene duplication and swapping, whereas class 3 inteins have a protease origin [10,14,24,42].

Proteins fold into various defined three-dimensional structures to carry out their unique biochemical functions. Proteins with similar structures and functions across different organisms share common ancestors and have evolved through divergent evolution [36]. However, protein structures could also converge into a similar structure to function analogously but having evolved from different ancestors. This convergent evolution is best exemplified by the catalytic Ser-His-Asp triad commonly found in hydrolases, suggesting the importance of structural and functional constraints required for specific catalysis [35,46,47]. Even though convergent evolution is a commonly observed phenomenon across the diversity of living organisms, the convergent evolution of protein structures has been documented only for small structural elements of proteins [48]. Structural convergence of an entire protein fold has not been identified [49]. The distinct mechanisms in protein splicing and newly identified possible ancestral domains might imply that class 3 inteins might have emerged via different evolutionary pathways or different ancestral proteins rather than divergent evolution from class 1 and 2 inteins. Despite the possible differences in the mechanism, class 3 inteins still have the same HINT fold presumably because the HINT fold could be an effective structural and functional solution for the protein-splicing reaction.

In summary, we determined the high-resolution crystal structures of two variants of class 3 *Mch*DnaB1 inteins and the engineered gp41-1 intein with the class 3 WCT motif. The three-dimensional structures, MD simulation, and biochemical data indicated a possible protein-splicing mechanism of class 3 inteins different from that of class 1 and 2 inteins. The protein-splicing mechanisms with diverse amino-acid types at the active sites cannot explain the divergent evolution model of class 3 inteins directly from class 1 and 2 inteins by random mutations. With the divergent evolution model, inteins would require several concurrent compensatory mutations for their survival what is a very unlike event (Figure 6). The high diversity of the active-site residue combinations of inteins might be reminiscent of independent evolutionary pathways originating from distantly related ancestral proteins such as proteases and translation initiation factors. Despite the different splicing mechanisms with various combinations of amino-acid types at the active sites, all splicing domains share the same HINT fold, which might suggest the convergence of the HINT fold possibly via different evolutionary pathways from distantly related origins.

## 4. Methods

### 4.1. Cloning of Class 3 Intein Expression Vectors

The gene encoding the *Mch*DnaB1 intein was amplified from the genomic DNA of *Mycobacterium chimaera* strain DSM 44,623 using the two oligonucleotides HB095: 5′–GTGGATCCGTCGGGAAGGCCCTTGC and HB096: 5′–CTGGGTACCTAGCGTGGAATTGTGCGTCG. The amplified gene was cloned between the *Bam*HI and *Kpn*I sites of pSKDuet16 [50], resulting in pHBDuet071 for *cis*-splicing tests. The gene was further PCR-amplified from pHBDuet071 using the two oligonucleotides J765: 5′–GAACAGATTGGTGGATCCGTCGGGAAGGCCCTTGC and J759: 5′–GTGCGGCCGCAAGCTTAATTGTGCGTCGGCACCATCCCGC for *Mch*DnaB1_HN, or J765 and J760: 5′–GTGCGGCCGCAAGCTTAGGCAGCGTGCGTCGGCACCATCCCGCG for *Mch*DnaB1_HAA. The PCR products were ligated into *Bam*HI and *Hin*dIII-digested pHYRSF53 [51], resulting in pHBRSF073 (*Mch*DnaB1_HN) and pHBRSF074 (*Mch*DnaB1_HAA) for the bacterial expression of N-terminally hexahistidine-tagged and SUMO-fused *Mch*DnaB1 intein variants.

Cys1Ala, Phe65Trp, and Asp107Cys mutations were introduced into the gp41-1 intein coding sequence via assembly PCR from plasmid pBHDuet37 [30] using the oligonucleotides HB019: 5′–CAAAACCTACACCGTAACGGAAGGATCCGGCTATGCGCTGGATCTGAAAACGCAGGTGC and HB015: 5′–CGGTCTGGGTCGGCCACAGATGTTCTTCGCTACAAATAATTTCTTTG, HB016: 5′–CGAAGAACATCTGTGGCCGACCCAGACCGGCGAAATG and HB017: 5′–CGCTCACTTCAATGCAGATCAGTTCGCGTTCATCCAGCTC, HB018: 5′–GAACGCGAACTGATCTGCATTGAAGTGAGCGGTAACCATCTG and HB014: 5′–CGTTCAGGATAAGTTTGTACTGGGTACCGCTCGAGCTGTTGTGGGTCAGAATGTCGTTC, thereby attaching the 3-residue N- and C-terminal junction sequences. The assembled PCR product was ligated into pBHDuet37 [30] using the *Bam*HI and *Kpn*I restriction sites, resulting in plasmid pHBDuet024 for *cis*-splicing tests. Variants encoding only Phe65Trp and Asp107Cys mutations (pHBDuet023) were generated the same way, but using HB013: 5′–CAAAACCTACACCGTAACGGAAGGATCCGGCTATTGCCTGGATCTGAAAACGCAGGTG instead of HB019. For introducing the Cys1Ala mutation (pHBDuet022), the oligonucleotides HB019 and HB014 were used. Plasmid pHBDuet021 was used as gp41-1 *cis*-splicing wild-type control [30]. For structural studies on gp41-1_WCT, the gene encoding the protein sequence was amplified from pHBDuet024 using the oligonucleotides I521: 5′–TTGGATCCGGTGGTGCCCTGGATCTGAAAACGCAG and I522: 5′–GTCAAGCTTAGTTGTGGGTCAGAATGTCGTTC and ligated into *Bam*HI and *Hin*dIII-digested pHYRSF53 [51] resulting in pHBRSF044 encoding N-terminally hexahistidine-tagged and SUMO-fused gp41-1_WCT. Plasmid pET22b_TRX_MSM encoding the *Msm*DnaB1 intein was a kind gift from Dr. FB. Perler (New England Biolabs, USA). The intein gene encoding the protein sequence was amplified using the oligonucleotides HK960: 5′–AGGGATCCGGTAAAGCACTGGCACTGGAT and HK961: 5′–AGCAAGCTTAGGTCGCATTATGGGTCGGAACCATACC and ligated into *Bam*HI and *Hin*dIII-digested pHYRSF53 [51] resulting in pCARSF64, encoding the N-terminally hexahistidine-tagged and SUMO-fused *Msm*DnaB1_HNAT with three N-terminal Ser-Gly-Lys, and two C-terminal Ala-Thr extein residues. Alternatively, HK960 was used with HK971: 5′–AGCAAGCTTAATTATGGGTCGGAACCATACC, resulting in pCARSF63-65 lacking the C-terminal extein sequence. pCARSF63-65 was used for the production of ^15^N-labeled *Msm*DnaB1. The *cis*-splicing vector pHBDuet060 was constructed by PCR amplification of the *Msm*DnaB1 intein from pCARSF64 using the oligonucleotides HB078: 5′–GGAAGGATCCGTGGGTAAGGCGCTCGCGCTCGACAC and HB079: 5′–ACTGGGTACCGAGTGTCGAGTTGTGCGTGGGAACCATG and ligation of the product into pSKDuet16 [50] using *Bam*HI and *Kpn*I sites.

The gene encoding the *Dra*Snf2 intein was amplified from the genomic *Deinococcus radiodurans* DNA (DSM-20539) using the oligonucleotides HB020: 5′–GAAGGATCCCTGGGCAAGGCGCAGC and HB021: 5′–ACTGGGTACCTTGCAGCGTGTTGTGGGTG including three residues of N- and C-terminal junction sequence. The PCR product was ligated into pSKDuet16 [50] using *Bam*HI and *Kpn*I sites, resulting in the *cis*-splicing vector pHBDuet027. The nested endonuclease domain was deleted by PCR amplification of the N- and C-terminal halves using HB020 and HB072: 5′–CGCTGCCGCCGCTGCCACTGCCACCGCTGCCACTACCGCCGGGGTCGAGGGGCAG, and HB071: 5′–CGGTGGCAGTGGCAGCGGCGGCAGCGGTGGCAGTGGCAGCGGCGGCGAGAAGAAAACG and SZ015: 5′–TGCCAAGCTTATTCCGTTACGGTG and assembled with HB020 and SZ015. The product was ligated into pSKDuet16 [50] as described above, resulting in the *cis*-splicing vector pHBDuet058 encoding the *Dra*Snf2 intein with a deletion of residues 121–266 replaced by an 18-residue GS-based linker (*Dra*Snf2^Δ128^). For deleting residues 121–251 (*Dra*Snf2^Δ131^, pHBDuet057), the oligonucleotides HB069: 5′–GCGGGCCACCCCGCCGGGGTCGAGGGGCAG, and HB070: 5′–CTGCCCCTCGACCCCGGCGGGGTGGCCCGCATTC were used instead of HB072 and HB071. For testing salt-inducible N-cleavage of a class 1 intein, plasmid pSADuet735 was used encoding the *Hut*MCM2 intein with the terminal and +1 intein residues mutated to Ala, flanked by two GB1 domains and N-terminal hexahistidine tag (H_6_-GB1-*Hut*MCM2_HAA-GB1) [28]. All the plasmids used, except for pSADuet735, are deposited at www.addgene.org (www.addgene.org/Hideo_Iwai).

### 4.2. Expression and Purification of MchDnaB1_HN, MchDnaB1_HAA, and gp41-1_WCT

Proteins were expressed in *E. coli* T7 Express strain (New England Biolabs, Ipswich, USA) using 2 L of LB medium supplemented with kanamycin by induction with a final concentration of 1 mM isopropyl-β-D-thiogalactoside (IPTG). *Mch*DnaB1_HAA and gp41-1_WCT were expressed at 37 °C for 3 h. *Mch*DnaB1_HN was expressed at 16 °C overnight. Induced cells were harvested by centrifugation at 4700× *g* for 10 min at 4 °C and frozen in liquid nitrogen for storage at −80 °C. Harvested cells were lysed in buffer A (50 mM sodium phosphate pH 8.0, 300 mM NaCl) using continuous passaging through an EmulsiFlex-C3 homogenizer (Avestin, Mannheim, Germany) at 15,000 psi for 10 min, 4 °C. Lysates were cleared by centrifugation at 38,000× *g* for 60 min, 4 °C. Proteins were purified in two steps by immobilized metal chelate affinity chromatography (IMAC) using 5 mL HisTrap FF columns (GE Healthcare, Chicago, Illinois, USA) as previously described, including the removal of the hexahistidine tag and SUMO fusion [51]. After each IMAC purification, proteins were dialyzed against the following buffers: *Mch*DnaB1_HN, buffer B (phosphate buffer saline (PBS) supplemented with 100 mM NaCl, 1 mM dithiothreitol (DTT)) and Buffer C (20 mM Tris-HCl pH 8.0, 200 mM NaCl, 1 mM DTT); *Mch*DnaB1_HAA, PBS and Buffer D (10 mM Tris-HCl pH 7.5, 100 mM NaCl, 1 mM DTT); gp41-1_WCT, PBS and deionized water. *Mch*DnaB1_HN and gp41-1_WCT were further purified using a Superdex^®^ 75 10/300 column (GE Healthcare, Chicago, IL, USA) in buffer E (10 mM Tris-HCl pH 8.0, 200 mM NaCl, 1 mM DTT) and Buffer F (0.5× PBS, 1 mM DTT), respectively. Peak fractions containing pure proteins were combined, and gp41-1_WCT was dialyzed against deionized water. Subsequently, the samples were concentrated using Macrosep^®^ Advance Centrifugal Devices 3K MWCO (Pall, Port Washington, DC, USA), and used for crystallization trials.

### 4.3. Proteolytic Inhibition Assays

The class 3 intein constructs H_6_-SUMO-*Mch*DnaB_HN and H_6_-SUMO-*Mch*DnaB1_HAA, and the salt-inducible class 1 intein H_6_-GB1-*Hut*MCM2_HAA-GB1 were expressed in *E. coli* T7 Express strain (New England Biolabs, Ipswich, MA, USA) at 37 °C in 5 mL LB medium containing 25 µg mL^−1^ kanamycin by induction with 1 mM IPTG for 3 h. Induced cells were harvested by centrifugation at 4700× *g* for 10 min, and proteins were purified by IMAC using Ni-NTA spin columns (QIAGEN, Hilden, Germany). For the proteolytic inhibition assays, the his-tagged SUMO fusion proteins containing the class 3 intein variants were eluted in 100 µL elution buffer (50 mM sodium phosphate pH 8.0, 300 mM NaCl, 250 mM imidazole) and immediately incubated after addition of a final concentration of 1 or 10 mM phenylmethanesulfonyl fluoride (PMSF) (Roche, Basel, Switzerland), 1 mM H_2_O_2_ (Sigma Aldrich, Steinheim, Germany), or 1 tablet/25 mL cOmplete™ Mini EDTA-free protease inhibitor cocktail (Roche, Basel, Switzerland) at room temperature (RT). The N-cleavable salt-inducible class 1 intein was incubated in 0.35 M sodium phosphate buffer pH 7.0, 3.5 M NaCl, 0.5 mM EDTA at RT. Samples were taken at 0-, 4-, 8-, 24-h points and analyzed by SDS-PAGE (16.5%). Band intensities were quantified using ImageJ 2.0.0-rc-69/1.52p. The quantification of the N-cleavage of H_6_-SUMO-*Mch*DnaB1_HN and H_6_-GB1-*Hut*MCM2_HAA-GB1 was derived from the equation, 100 × [(CP/(CP+P))_t_ − (CP/(CP+P))_t0_]/[1 − (CP/(CP+P))_t0_], where CP is the sum of the cleavage products (H_6_-SUMO and *Mch*DnaB1_HN, or H_6_-GB1 and *Hut*MCM2_HAA-GB1), t and t_0_ are the time and zero-time points, and P is the unreacted precursor.

### 4.4. Protein Cis-Splicing Tests

To assay protein *cis*-splicing, the vectors encoding the inteins *Msm*DnaB1 (pHBDuet060), *Mch*DnaB1 (pHBDuet071), *Dra*Snf2 (pHBDuet027), *Dra*Snf2^Δ128^ (pHBDuet058), and *Dra*Snf2^Δ131^ (pHBDuet057) were expressed in *E. coli* T7 Express strain (New England Biolabs, Ipswich, MA, USA) as described in the section “Proteolytic inhibition assays”. Proteins were purified and analyzed as described above.

### 4.5. Crystallization and Structure Determination of MchDnaB1_HN, MchDnaB1_HAA, gp41-1_WCT

Diffracting crystals of *Mch*DnaB1_HN were obtained using the sitting drop vapor diffusion technique in 96 well-plates at room temperature by mixing 100 nL concentrated protein (13.4 mg/mL) with 100 nL mother liquid (100 mM Tris-HCl pH 9, 200 mM MgCl_2_, 30% (*w*/*v*) polyethylene glycol (PEG) 4000). Data were collected at 100 K under cryo-stream using a flash frozen crystal by liquid nitrogen without additional cryo-protectant using the beamline I03 at Diamond Light Source (DLS, Didcot, UK) equipped with a Pilatus detector (Pilatus3 6M). Data were processed to 1.66 Å (Supplemental Table S1). The structure was solved by molecular replacement using PHASER [52] with the *Msm*DnaB1 intein (6bs8) as a search model [18]. The model was built using PHENIX [53], AutoBuild [54], manually corrected with COOT [55], and refined using PHENIX [53]. We also used AutoBuild because we expected reliable model building due to the high-resolution data and compared the structure of loop regions with manual building. The final model consists of two molecules in the asymmetric unit. The four residues of the sequence SVGK preceding Ala1 of the intein were clearly missing in the electron density. A loop region between residues Ser91-Leu104 (chain A) and Gly90-Leu104 (chain B) was not modeled due to insufficient density information. The electron density for the side chain Cys124 suggested that it was oxidized and was therefore modeled as S-oxy cysteine (Csx). Alternate conformations were modeled for Thr15, Asp19, Arg46, and Csx124 (chain A) and Cys124 and His144 (chain B). The final model includes one Cl^−^ ion originating from the crystallization buffer. The structure was validated using MolProbity (score 1.07, 100th percentile) [56].

*Mch*DnaB1_HAA crystals were obtained as described above using concentrated protein (13 mg/mL) after adjusting the DTT concentration to 10 mM and mother liquor (100 mM Tris-HCl pH 7.5, 200 mM MgCl_2_, 25% (*w*/*v*) PEG 4000). Crystals flash-frozen by liquid nitrogen were shipped and collected at the fully automated beamline ID30A-1/MASSIF-1 [57,58,59] at ESRF (Grenoble, France) equipped with a Pilatus detector (Pilatus3 2M) and processed to 1.63 Å (Supplemental Table S1). The structure was solved by molecular replacement using PHASER [52] with the *Mch*DnaB1_HN structure as a search model. The structure model was built using ARP/wARP [60], manually corrected using COOT [55], and refined using PHENIX [53]. We used ARP/wARP [60] due to the similar reason as Autobuild for the structure of loop regions. The final model consists of two molecules in the asymmetric unit. Four residues of the sequence SVGK preceding Ala1 of the intein were clearly missing in the electron density. A loop region between residues Gly90-Leu105 (chain A) and Gly90-Leu104 (chain B) was not modeled due to the lack of electron densities. Alternate conformations were modeled for Thr15, Pro142, (chain A), and Val87 (chain B). The final model contains one Cl^-^ ion. The structure was validated using MolProbity (score 1.04, 100th percentile) and PDB_REDO [56,61].

Diffracting crystals of gp41-1_WCT were obtained as above with a protein concentration of 40 mg/mL and mother liquor (100 mM bis-tris pH 5.5, 200 mM (NH_4_)_2_SO_4_), 25% (*w*/*v*) PEG 3350). Data were collected at beamline I04 at DLS (Didcot, UK) equipped with a Pilatus detector (PILATUS 6M-F) and 1.85 Å (Supplemental Table S1). The structure was solved by molecular replacement using PHASER with the gp41-1 intein (6qaz) as a search model [30]. The structure model was built using PHENIX AutoBuild [54], manually corrected with COOT [55], and refined using PHENIX [53]. The entire protein chain (one molecule in the asymmetric unit) could be traced in the electron density without breaks for all 128 residues except for the first Ser residue. A non-canonical *cis* peptide bond was modeled between Lys87 and Glu88, which is also found in the search model. Alternate conformations were modeled for Leu25, Ser28, Val38, and Ser46. Additional density was observed for the side-chain of Cys83, indicating oxidation and was modeled as 3-sulfinoalanine (Csd). The structure was validated using MolProbity (score 1.28, 99th percentile) [56].

### 4.6. Molecular Dynamics Simulation

We performed MD simulations of the three different proteins, *Mch*DnaB1_HN, *Mch*DnaB1_HAA, and gp41-1_WCT, with and without modeling an N-extein. In the crystal structures of both *Mch*DnaB1_HN (chain B) and *Mch*DnaB1_HAA (chain A), residues 9–104 or 105 in the loop region were not modelled (see above). We modelled these missing residues with MODELLER software [62], and used them as the starting model for the simulation without the N-terminal residues. The four-residue N-extein (“SVGK”) was also modeled on the structure to generate the initial structure for the MD simulation with the N-terminal residues using the MODELLER software [62]. The crystal structure of gp41-1_WCT (6riz) contained all the residues, including the N-extein part of the “GG” sequence, and it was used as the starting model for the simulation with the N-extein part. The initial structure of the gp41-1_WCT simulation without the N-extein fragment was derived by removing the first two glycine residues from the crystal structure.

The MD simulations were performed using Gromacs 2018 software [63] and Amber ff99SB-ILDN force field [64] in a rectangular simulation box with periodic boundary conditions. The protein coordinates from the crystal structures of *Mch*DnaB1_HN, *Mch*DnaB1_HAA, and gp41-1_WCT were solvated with approximately 11,000 and 7500 TIP3P water molecules [65], and the systems were made electroneutral by adding an appropriate number of Na^+^ ions. The structures were first energy minimized for 1000 steps with the steepest descent algorithm. The production simulations were run for 400 ns with a timestep of 2 fs for each system. All bond lengths were constrained with LINCS [66]. The temperature was set to 303 K with the v-rescale thermostat [67], and Parrinello–Rahman barostat was used for isotropic pressure coupling at 1 bar [68]. Electrostatic interactions were treated with particle mesh Ewald [68,69], and Lennard–Jones interaction cut-off was set to 1.0 nm. The χ^1^ angle of the cysteine residue within the active site (Cys124 for *Mch*DnaB1_HN and *Mch*DnaB1_HAA, and Cys107 for gp41-1_WCT, respectively) was analyzed with Gromacs utilities. The simulation data are available from the Zenodo repository (DOI:10.5281/zenodo.3448608).

## Supplementary Materials

The following are available online at https://www.mdpi.com/1422-0067/21/21/8367/s1, Table S1: Data collection and refinement statistics, Table S2: Structural homology identified by DALI server, Figure S1: Comparison of different class 3 inteins, Figure S2: The crystal structures of the *Mch*DnaB1 intein variants, Figure S3: N-cleavage of class-3 *Mch*DnaB1 intein variants, Figure S4: Inhibition of N-cleavage of the class-1 *Hut*MCM2_HAA intein by H_2_O_2_, Figure S5: Proposed reaction steps f or the protein splicing mechanism catalyzed by the class 3 intein and the relations to the solved crystal structures, Figure S6: Analysis of χ^1^ angles of the catalytic-triad residues (Cys in black, His in red, and Thr in green) during 400-nsec MD simulations of the two variants of the MchDnaB1 intein (*Mch*DnaB1_HN and *Mch*DnaB1_HAA) and the engineered gp41-1 intein with WCT motif (gp41-1_WCT), Figure S7: Possible ancestral domains of the HINT fold.

## Abbreviations

BIL	Bacterial Intein-Like
HINT	Hedgehog/INTein
Hh-C	the C-terminal domain of the Hedgehog protein or hog protein
IMAC	immobilized metal affinity chromatography
IPTG	isopropyl-β-D-thiogalactoside
MchDnaB1 intein	DnaB1 intein from Mycobacterium chimaera
PDB	Protein Data Bank
r.m.s.d.	root-mean-square deviation
HSQC	heteronuclear single quantum correlation
PEG	polyethylene glycol
PMSF	phenylmethanesulfonyl fluoride
DTT	dithiothreitol.
