# Supplementary material for: The Convergence of the Hedgehog/Intein Fold in Different Protein Splicing Mechanisms
Source: Int J Mol Sci. 2020 Nov 7;21(21):8367. doi: 10.3390/ijms21218367 (PMC7664689; doi:10.3390/ijms21218367)
Supplement: Supplementary file 1 [file ijms-21-08367-s001.pdf]

**Supplemental Table S1** Data collection and refinement statistics.

| | | | |
|---|---|---|---|
| **Data collection** | | | |
| Intein | *Mch*DnaB1_HN | *Mch*DnaB1_HAA | gp41-1_WCT |
| Beamline | i03 (Diamond) | ID30A-1 (ESRF) | i04 (Diamond) |
| Program | HKL3000 | Mosflm | HKL3000 |
| Wavelength | 0.9763 | 0.966 | 0.9795 |
| Space group | $P2_1$ | $P2_1$ | $P6_522$ |
| Molecules / a.u. | 2 | 2 | 1 |
| Unit cell *a*, *b*, *c* (Å); | 31.25, 95.34, 35.79 | 31.31, 95.78, 35.86 | 90.22, 90.22, 71.73 |
| $\alpha$, $\beta$, $\gamma$ (°) | 90, 107.14, 90 | 90, 107.37, 90 | 90, 90, 120 |
| Resolution range (Å) | 50.0 - 1.66 (1.68 - 1.66) | 34.23 - 1.66 (1.66-1.63) | 50 - 1.85 (1.88 - 1.85) |
| Total no. of reflections | 135528 | 60056 | 509713 |
| No. of unique reflections | 22641 (731) | 24075 (1195) | 15226 (741) |
| $R_{merge}$ (%)† | 8.5 (36.7) | 8.1 (50.1) | 11.7 (77.3) |
| $<I/\sigma_I>$ | 18.7 (2.2) | 7.3 (1.9) | 33 (2.7) |
| $CC_{1/2}$& | 0.993 (0.543) | 0.994 (0.603) | 1.000 (0.554) |
| Completeness (%) | 95.0 (60.2) | 95.8 (96.8) | 100 (99.1) |
| Redundancy | 6.0 (2.7) | 2.5 (2.6) | 33.5 (14.3) |
| **Refinement** | | | |
| Resolution range (Å) | 47.67 - 1.66 (1.74 - 1.66) | 34.23- 1.63 (1.70 - 1.63) | 45.11 - 1.85 (1.99 - 1.85) |
| No. of reflections (refinement / $R_{free}$) | 21521 / 1037 | 24035 / 1186 | 15147 / 717 |
| $R$ / $R_{free}$ (%)‡ | 15.9/ 21.4 | 17.0 / 21.7 | 16.9/ 20.2 |
| No. atoms | | | |
| Protein | 1954 | 1920 | 1028 |
| Ion | 1 | 1 | 0 |
| Water | 220 | 268 | 157 |
| R.m.s. deviations from ideal | | | |
| Bond lengths (Å) | 0.006 | 0.006 | 0.007 |
| Bond angles (°) | 0.85 | 0.81 | 0.91 |
| Ramachandran plot | | | |
| Favored (%) | 98.0 | 96.8 | 98.4 |
| Outliers (%) | 0 | 0 | 0 |
| PDB code | 6rix | 6riy | 6riz |

The highest resolution shell is shown in parentheses.

†$R_{merge} = \sum_h\sum_i | I_i - \langle I \rangle | / \sum_h\sum_i I_i$, where $I_i$ is the observed intensity of the *i*-th measurement of reflection h, and $\langle I \rangle$ is the average intensity of that reflection obtained from multiple observations.

‡$R = \sum||Fo|-|Fc||/\sum|Fo|$, where Fo and Fc are the observed and calculated structure factors, respectively, calculated for all data. $R_{free}$ was defined in Brünger, Nature, 355, 472–475 (1992).

&$CC_{1/2}$ was defined in Karplus et al. Science, 336, 1030–1033 (2012).

**Supplemental Table S2** Structural homology identified by DALI server.

| Protein | PDB | rmsd | Z-score | Length | # of residues | Seq. identity (%) |
|---|---|---|---|---|---|---|
| Translation Initiation Factor 5 (IF-5) | 1BKB | 2.3 | 2.7 | 49 | 136 | 16 |
| Eukaryotic Translation Initiation factor 5A2 (IF-5A2) | 3HKS | 2.1 | 2.6 | 49 | 142 | 16 |
| Elongation Factor P (EF-P) | 1UEB | 2.2 | 2.5 | 44 | 184 | 11 |
| PI-*Sce*I | 1DFA | 2.2 | 8.9 | 124 | 429 | 19 |
| 17-hedgehog | 1AT0 | 1.9 | 17.0 | 128 | 145 | 16 |

**a**

| Class 3 | Block A | Block B | Block F | Block G |
|---|---|---|---|---|
| *MP-Be*DnaB | Q/PLALNTEVPTPSG | GTEITASASHGWTT | PVKCIGIDTEDHLFQ | SRILTHN/T |
| *Bvi*IcmO | F/PQPLHSLVRMADG | GRSVEAARVHHWPV | PARCLVVADEMHCYI | HDIVTHN/C |
| *Dra*Snf2 | K/AQPLDAKVLTPLG | GASVEADAEHLWNV | PAQCIAVDAPDHLYV | GYIVTHN/T |
| *Mle*DnaB | K/ALALDTPLPTPTG | GTVIVADAQHQWPT | PVRCVEVDNAAHLYL | GMVPTHN/S |
| *Msm*DnaB1 | K/ALALDTPLPTPSG | GTAIVADAQHQWPT | PVRCVEVDNPEHLYL | GMVPTHN/S |
| *Mch*DnaB1 | K/ALALYTPLPTPSG | GTVIVADAAHQWPT | PVRCVEVDNPAHLYL | GMVPTHN/S |
| **Class 1** | | | | |
| *Npu*DnaE | Y/CLSYETEILTVEY | GSVIRATSDHRFLT | NVYDIGVER-DHNFA | NGFIASN/C |
| *Npu*DnaB | G/CLAGDSLVTLVDS | GRKIRATGNHKFLT | EVFDLTVPG-LHNFV | NNIIVHN/S |
| gp41-1 | Y/CLDLKTQVQTPQG | GKEIICSEEHLFPT | ELIDIEVSG-NHLFY | NDILTHN/S |

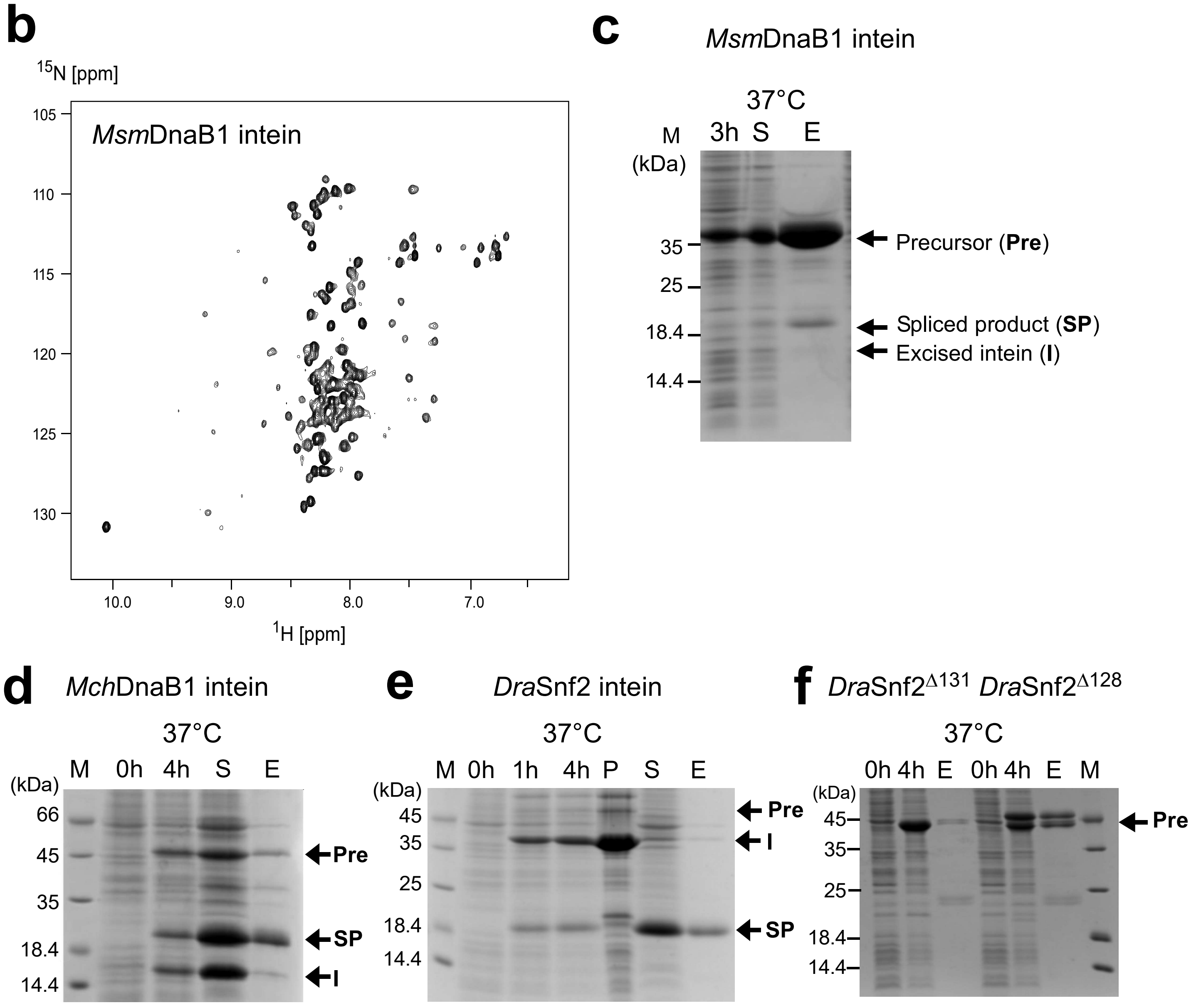


**Supplemental Fig. S1** Comparison of different class 3 inteins. (**a**) A sequence alignment of class 3 intens and class 1 inteins for blocks A, B, F, and G. (**b**) [$^1$H,$^{15}$N]-HSQC spectrum of *Msm*DnaB1_HAA showing poor dispersion of peaks. SDS-PAGE analysis of *cis*-splicing at 37 °C of (**c**) *Msm*DnaB1 intein, (**d**) *Mch*DnaB1 intein, (**e**) *Dra*Snf2 intein, and (**f**) *Dra*Snf2$^{\Delta131}$ and *Dra*Snf2$^{\Delta128,}$ in which 131 residues and 128 residues from the endonuclease domain of *Dra*Snf2 inteinwere removed. Pre, SP, and I indicate the positions for precursor proteins, spliced proteins, and inteins, respectively. 0h, 1h, and 4h show samples taken at 0 hour, 1 hour, and 4 hours after the induction, respectively. M, P, S, and E indicate molecular marker, pellet, supernatant, and elution from Ni-NTA columns, respectively.

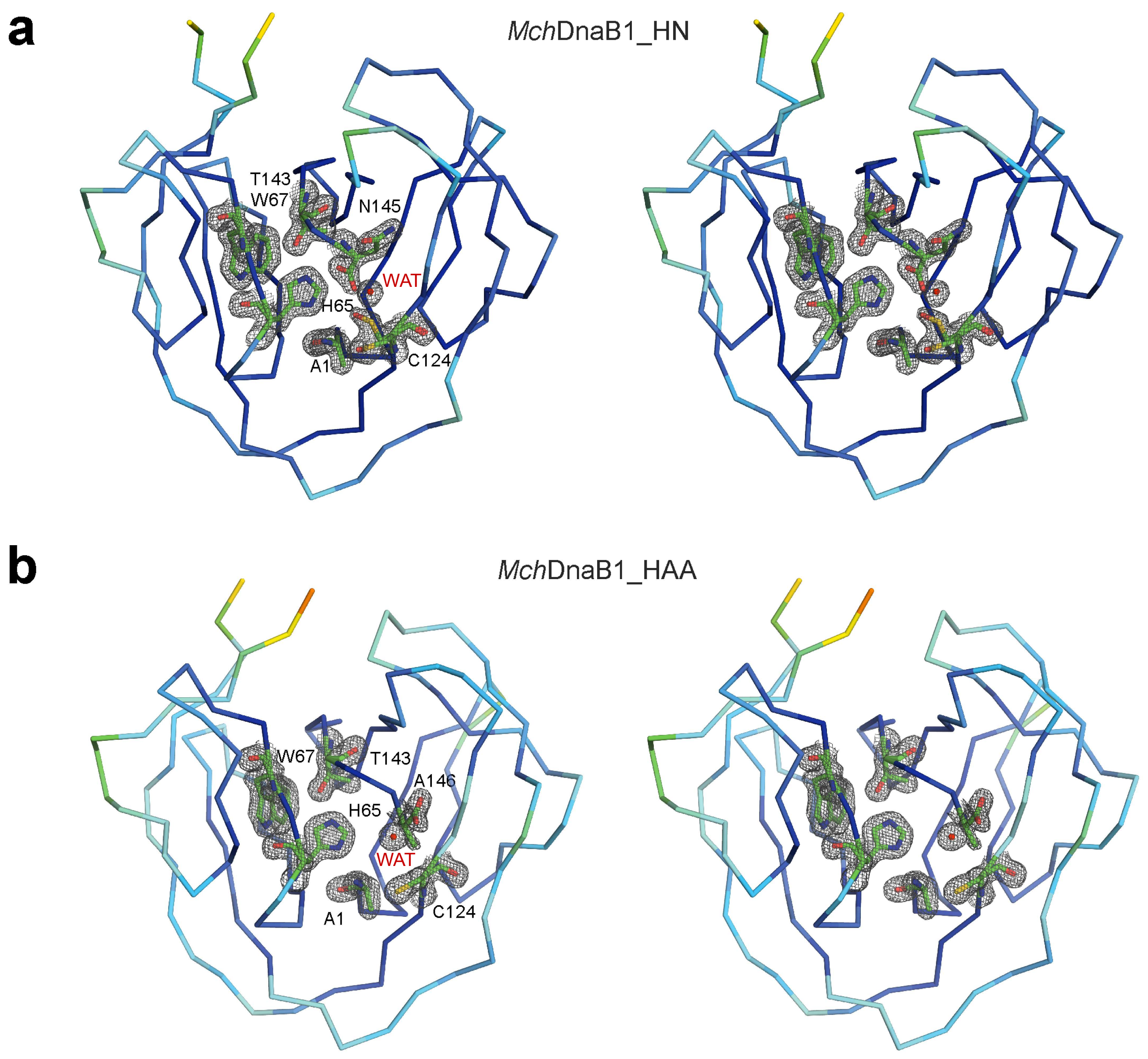


**Supplemental Fig. S2:** The crystal structures of the *Mch*DnaB1 intein variants. Stereo-views of the backbone structures of (**a**) *Mch*DnaB1_HN (chain A with oxidized Cys124) and (**b**) *Mch*DnaB1_HAA (chain A) showing the electron densities at the active-sites. WATs in red indicate the modeled oxyanion waters. The structures were depicted and colored according to the temperature factor using PyMol.

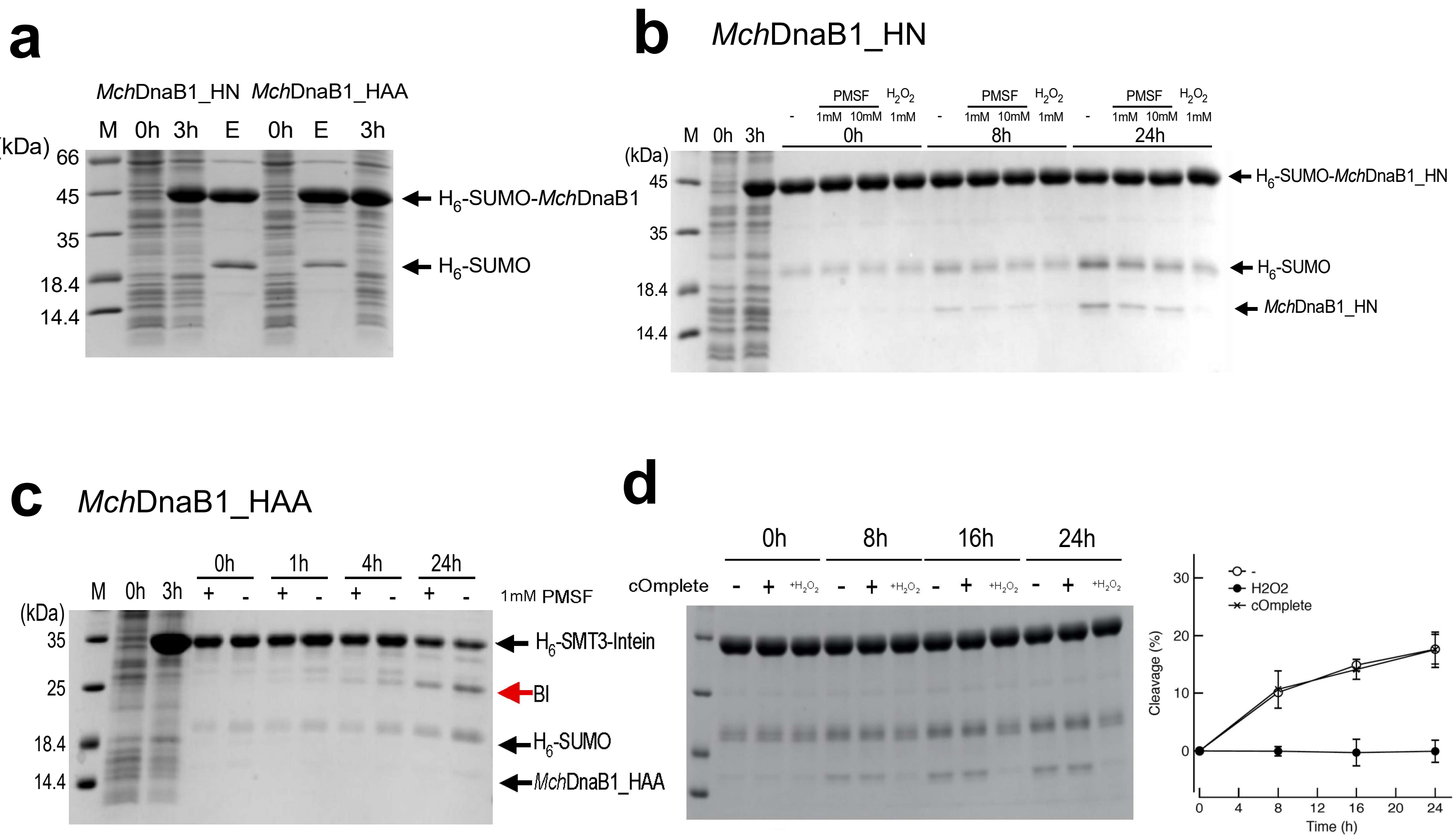


**Supplemental Fig. S3:** N-cleavage of class-3 *Mch*DnaB1 intein variants. (**a**) N-cleavage of *Mch*DnaB1_HN and *Mch*DnaB1_HAA inteins immediately after purification. (**b-c**) Inhibition of N-cleavage of *Mch*DnaB1_HN (**b**) and *Mch*DnaB1_HAA (**c**) by addition of the indicated concentrations of PMSF and $H_2O_2$ over time. (-), without inhibitor. (**d**) Inhibition with protease inhibitor cocktail (cOmplete). (**b-d**) After IMAC purification, the eluted protein was immediately incubated with the indicated inhibitors, and N-cleavage was monitored for 24 hours. Samples were analyzed by SDS-PAGE at the indicated time points. (**a-c**) Arrows indicate the corresponding bands for $H_6$-SUMO-intein, the precursor before cleavage, cleaved intein (*Mch*DnaB1_HN, or *Mch*DnaB1_HAA), and cleaved $H_6$-SUMO. BI stands for the branched intermediate. M, 0h, and 3h indicate molecular weight marker, the sample before induction, and 3 hours after protein induction, respectively.

**N-cleavage by salt-inducible class 1 intein ($H_6$-GB1-*Hut*MCM2_HAA-GB1)**

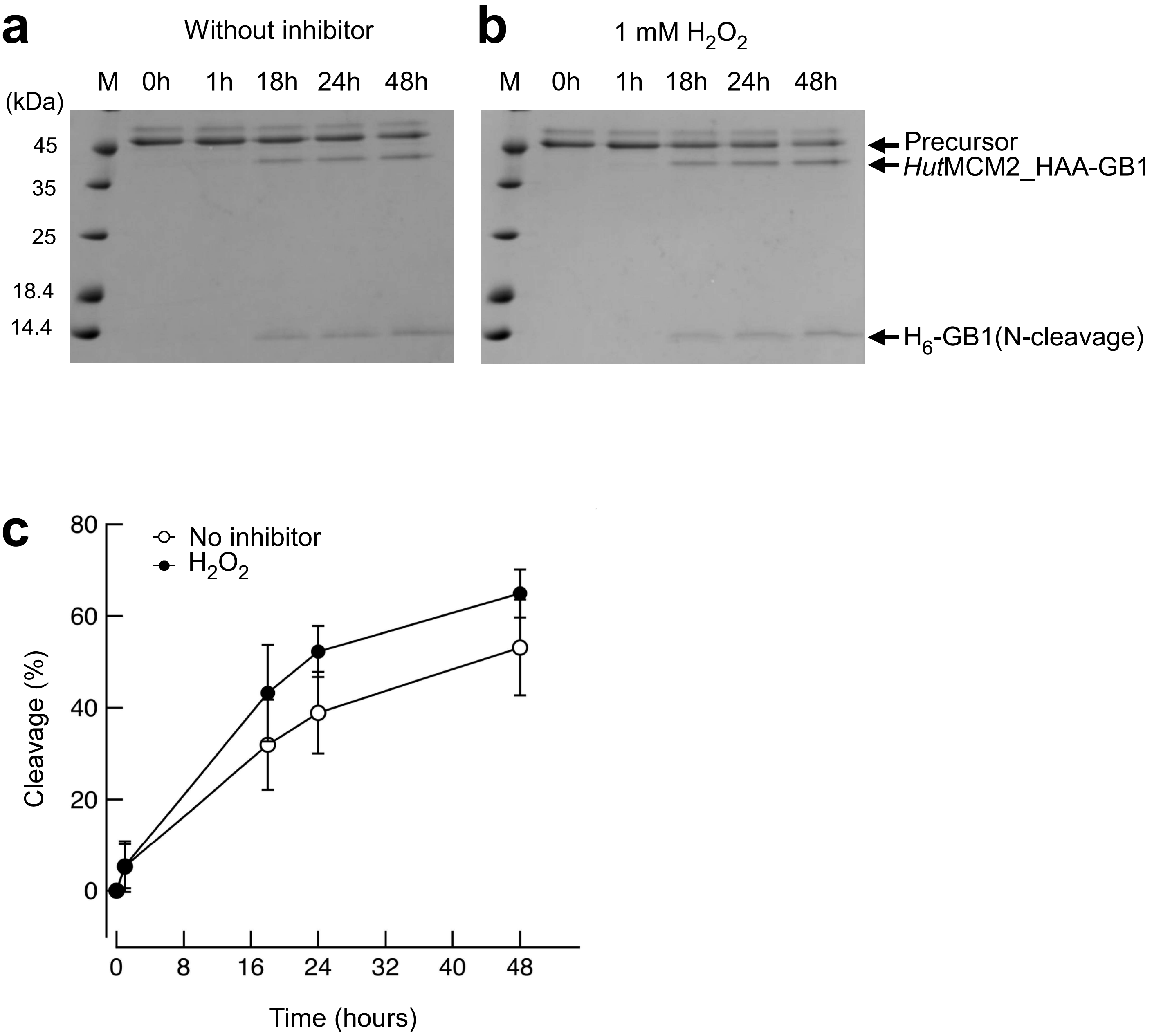


**Supplemental Fig. S4:** Inhibition of N-cleavage of the class-1 *Hut*MCM2_HAA intein by $H_2O_2$. After IMAC purification, the eluted protein was immediately incubated at high salinity (3.5 M NaCl) in the absence (**a**) or presence (**b**) of 1 mM $H_2O_2$. The N-cleavage was monitored for 48 hours by SDS-PAGE analysis. (**c**) Quantification of N-cleavage of the experiment in (**a**) and (**b**). Data were averaged from three individual experiments. Error bars represent the standard deviations.

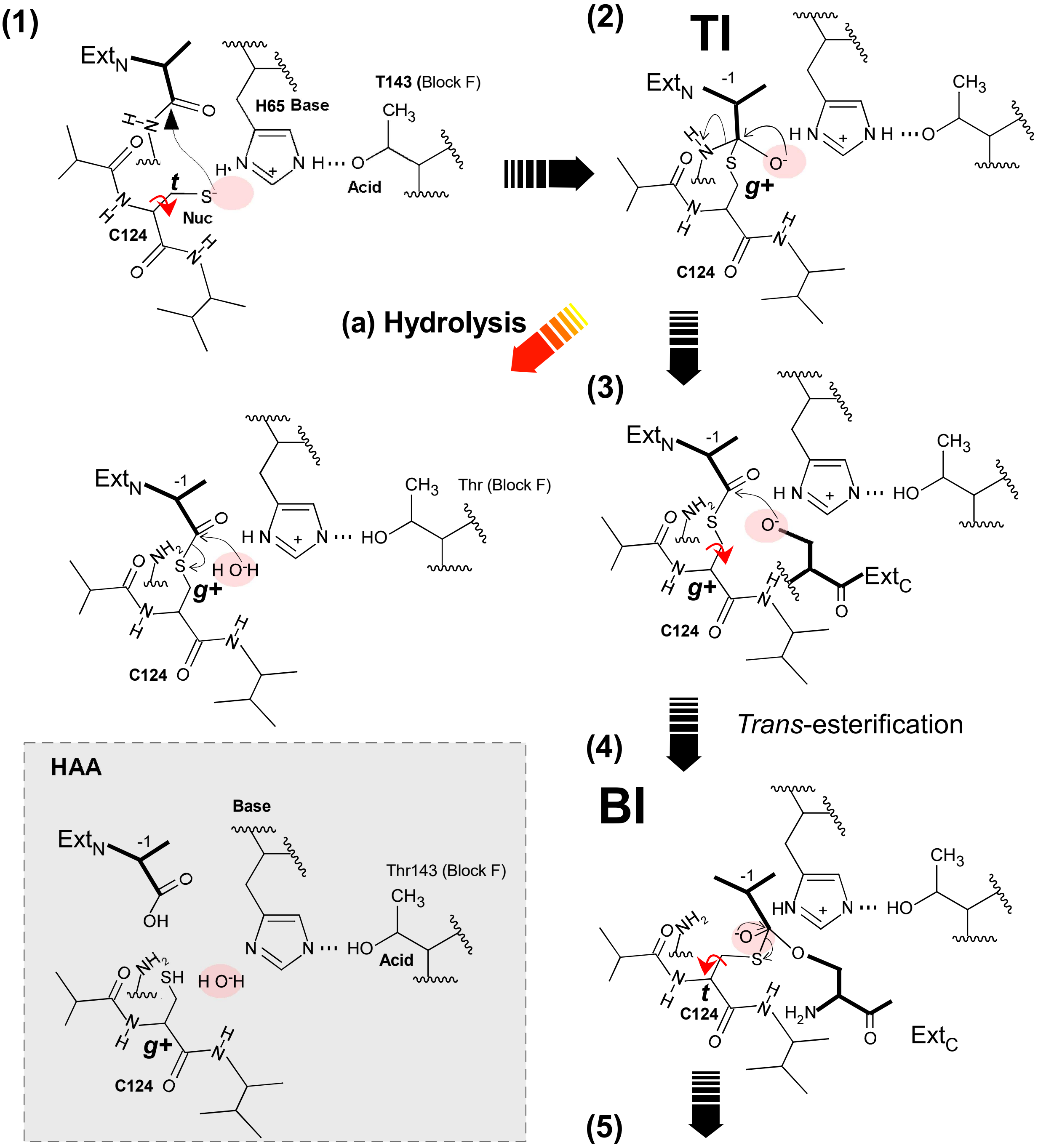


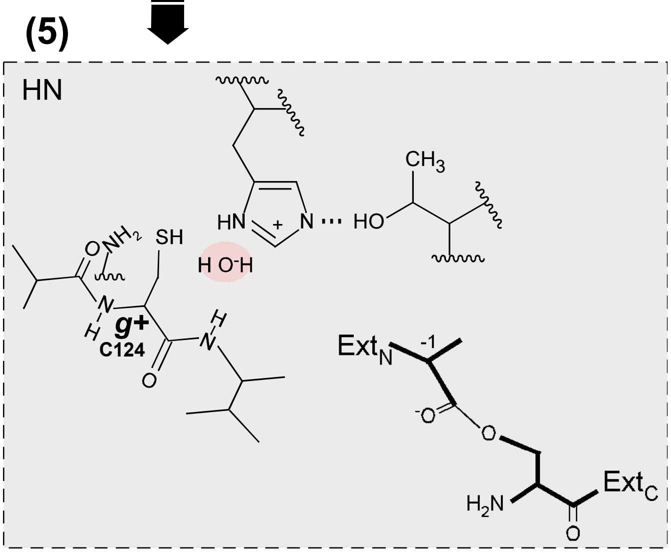


**Supplemental Fig. S5:** Proposed reaction steps for the protein splicing mechanism catalyzed by the class 3 intein and the relations to the solved crystal structures. (1) High energy ground state before splicing. (2) Tetrahedral Intermediate (TI) status after rotation of Cys124 to the gauche$^{+}$ conformation. (3) Branched Intermediate (BI) status. Rotation of Cys124 to the *trans* conformation will bring the thioester intermediate closer to the nucleophilic residue of the C-extein. (4) Trans-esterification step via a tetrahedral intermediate. Rotation of Cys124 back to the gauche$^{+}$ conformation. (5) Post-splicing status. Exteins are released from the intein. HN and HAA stand for the conformations represented by the crystal structures of *Mch*DnaB1_HN and *Mch*DnaB1_HAA, respectively. (a) The non-productive (off-pathway) N-cleavage (hydrolysis) is indicated by a red arrow.

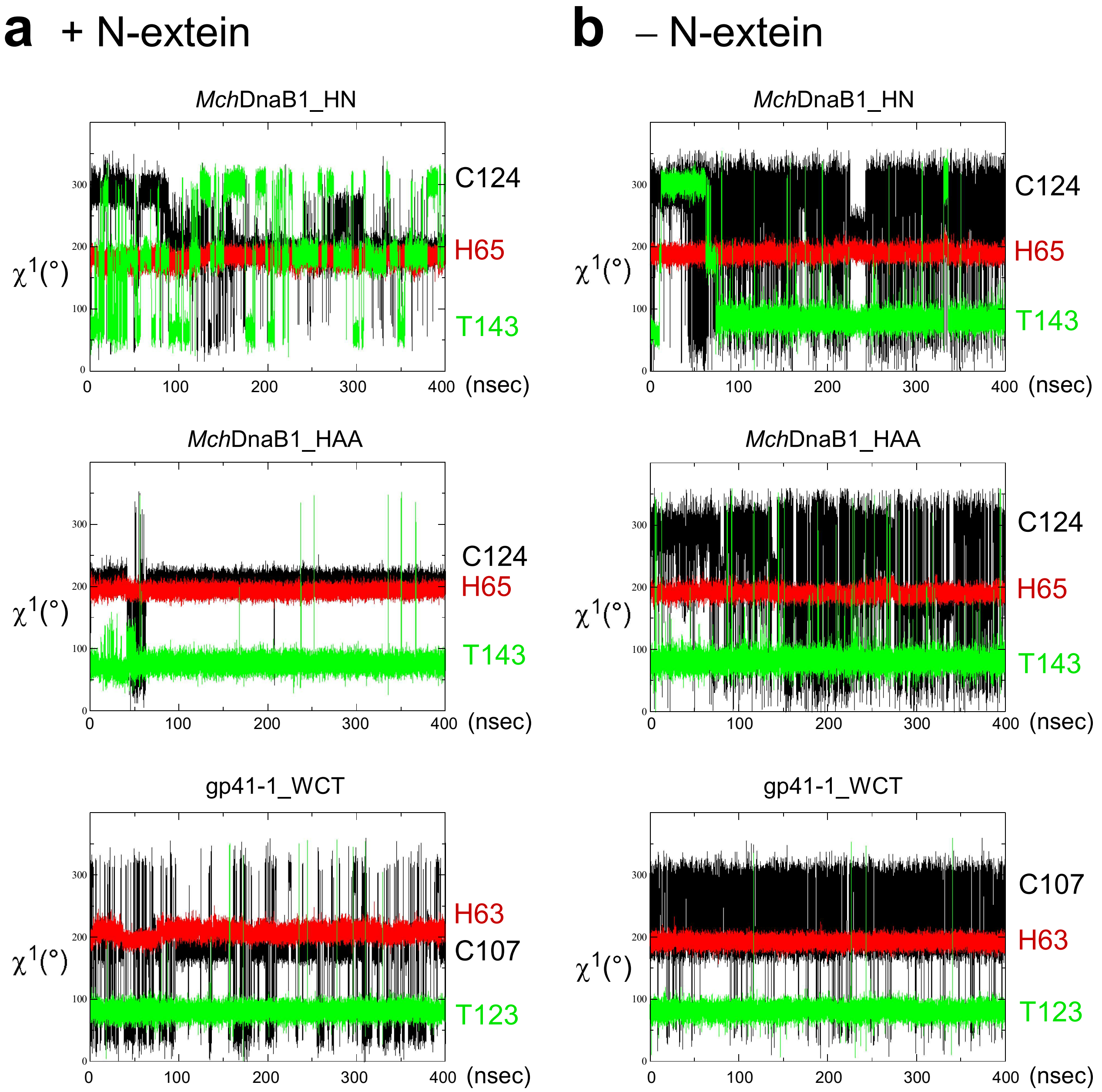


**Supplemental Fig. S6** Analysis of $\chi$1 angles of the catalytic-triad residues (Cys in black, His in red, and Thr in green) during 400-nsec MD simulations of the two variants of the *Mch*DnaB1 intein (*Mch*DnaB1_HN and *Mch*DnaB1_HAA) and the engineered gp41-1 intein with WCT motif (gp41-1_WCT). (**a**) Trajectories of the $\chi$1 angles of the catalytic-triad residues during the 400-nano sec MD simulation with the modeled N-extein sequence. (**b**) Trajectories of the same $\chi$1 angles without N-extein.

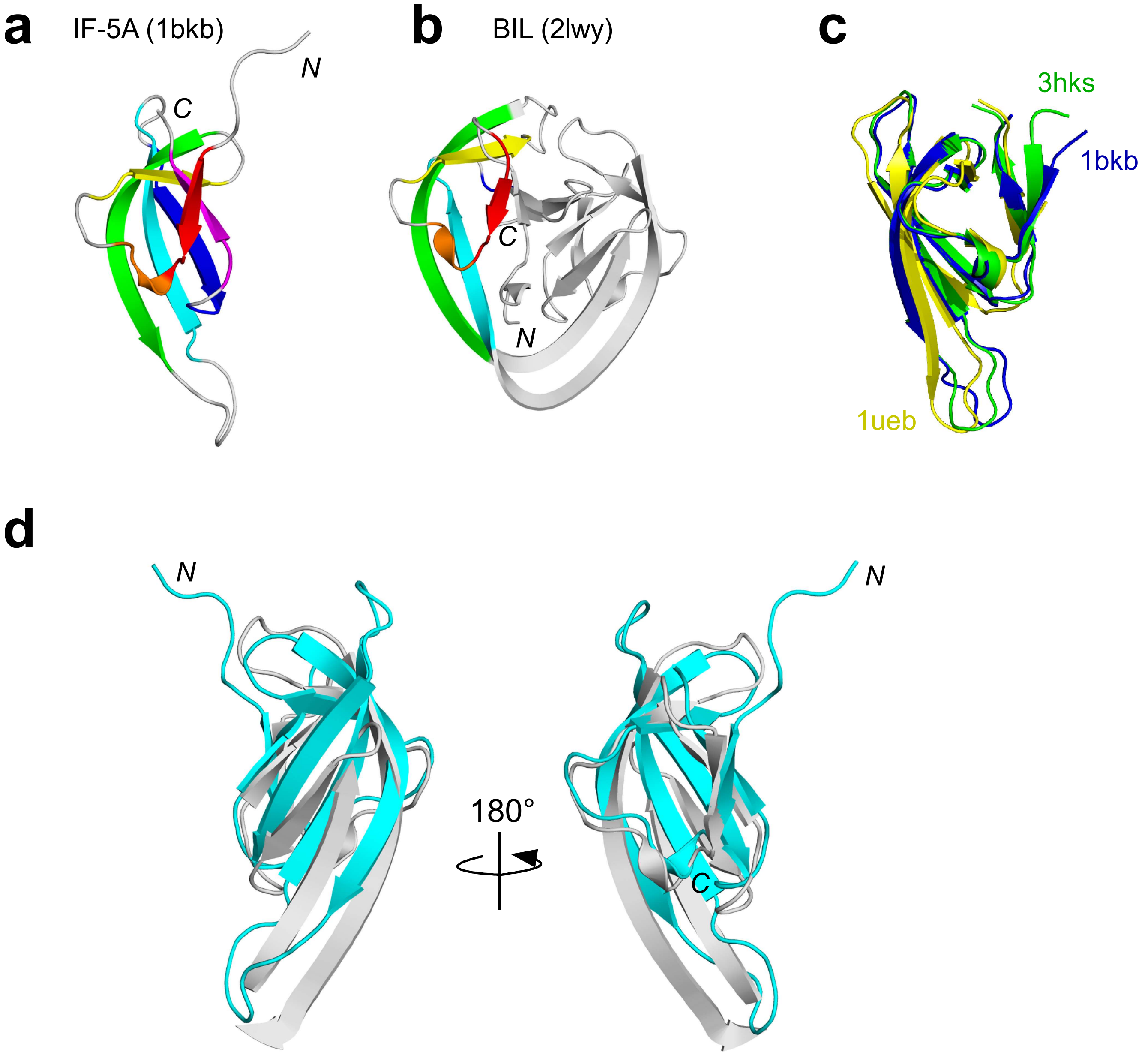


**Supplemental Fig. S7**: Possible ancestral domains of the HINT fold. (**a**) The N-terminal domain of IF-5A from *Pyrobaculum aerophilum* in the crystal structure (1bkb). The secondary structures used for the structural alignment are colored. (**b**) The crystal structure of the BIL4 domain from Clostridium thermocellum (2lwy). The secondary structures used for the superposition with the structure of IF-5A are colored with the same colors as in (a). (**c**) A superposition of the three crystal structures of Translation Initiation Factor 5 (IF-5A) from *Pyrobaculum aerophilum* (1bkb), Eukaryotic Translation Initiation factor 5A2 (3hks), and Elongation Factor P (1eub). (**d**) Overlays of the two structures from the N-terminal domain of IF-5A and *Cth*BIL4 domain. Only the regions of *Cth*BIL4 used for the superposition with the N-terminal domain of IF-5A are shown.
